# The geographic pattern of Belgian mortality: can socio-economic characteristics explain area differences?

**DOI:** 10.1186/s13690-016-0135-y

**Published:** 2016-06-08

**Authors:** Wanda M. J. Van Hemelrijck, Didier Willaert, Sylvie Gadeyne

**Affiliations:** Interface Demography, Department of Sociology, Vrije Universiteit Brussel, Pleinlaan 2, 1050 Brussels, Belgium

**Keywords:** Belgium, Mortality, Geographic distribution, Socio-economic position, Poisson regression, Multilevel

## Abstract

**Background:**

Country averages for health outcomes hide important within-country variations. This paper probes into the geographic Belgian pattern of all-cause mortality and wishes to investigate the contribution of individual and area socio-economic characteristics to geographic mortality differences in men aged 45–64 during the period 2001–2011.

**Methods:**

Data originate from a linkage between the Belgian census of 2001 and register data on mortality and emigration during the period 2001–2011. Mortality rate ratios (MRRs) are estimated for districts and sub-districts compared to the Belgian average mortality level using Poisson regression modelling. Individual socio-economic position (SEP) indicators are added to examine the impact of these characteristics on the observed geographic pattern. In order to scrutinize the contribution of area-level socio-economic characteristics, random intercepts Poisson modelling is performed with predictors at the individual and the sub-district level. Random intercepts and slopes models are fitted to explore variability of individual-level SEP effects.

**Results:**

All-cause MRRs for middle-aged Belgian men are higher in the geographic areas of the Walloon region and the Brussels-Capital Region (BCR) compared to those in the Flemish region. The highest MRRs are observed in the inner city of the BCR and in several Walloon cities. Their disadvantage can partially be explained by the lower individual SEP of men living in these areas. Similarly, the relatively low MRRs observed in the districts of Halle-Vilvoorde, Arlon and Virton can be related to the higher individual SEP. Among the area-level characteristics, both the percentage of men employed and the percentage of labourers in a sub-district have a protective effect on the individual MRR, regardless of individual SEP. Variability in individual-level SEP effects is limited.

**Conclusions:**

Individual SEP partly explains the observed mortality gap in Belgium for some areas. The percentage of men employed and the percentage of labourers in a sub-district have an additional effect on the individual MRR aside from that of individual SEP. However, these socio-economic factors cannot explain all of the observed differences. Other mechanisms such as public health policy, cultural habits and environmental influences contribute to the observed geographic pattern in all-cause mortality among middle-aged men.

**Electronic supplementary material:**

The online version of this article (doi:10.1186/s13690-016-0135-y) contains supplementary material, which is available to authorized users.

## Background

### Geographic patterns in health and mortality

Country averages for health outcomes hide substantial within-country variation [23, 54]. Geographic differences have been observed for a variety of health outcomes in several countries [10, 11, 17, 23, 32, 35, 39, 42, 45, 52, 53]. In Belgium, morbidity and mortality generally appear higher in the south (Walloon Region) than in the north (Flemish Region) of the country [13, 14, 37, 38, 48–50]. Renard et al. [38] map out cause-specific premature mortality (ages 1–74) for a number of major causes of death in Belgium. Maps show a clear north–south divide. The same is observed for cardiovascular, diabetes, alcohol-related, mental and neurological diseases, diabetes, and non-transport accident mortality. A high mortality zone stretching from the southeast to the northwest of the country is observed in head and neck cancers, suicide and to some extent road traffic accidents. A number of causes of death such as lung, colorectal and breast cancer mortality are much less geographically patterned.

### Socio-economic and geographic distributions

To explain geographic mortality differences, social scientists often refer to the differential distribution of individual socio-economic position (SEP) [7, 9, 10, 13, 14, 35]. The association between SEP and health or mortality has been well established since the publication of ‘The Black Report’ in 1981, leading to an explosion of research on the topic [29]. Generally, studies show a ‘social gradient’, namely that lower SEP is associated with higher mortality or worse health outcomes for the majority of mortality causes [2, 12–16, 21, 31, 36, 44, 47]. Traditional indicators for SEP are educational level, occupation and income, each implying material and non-material factors that could potentially affect health.

### Health and mortality: who you are and where you live

To explain health differentials, literature not only focuses on the abovementioned individual characteristics [1, 6, 18, 20, 26, 43, 46]. Effects of the social and physical environment are increasingly taken into account. Among these area effects, we can distinguish between collective effects and contextual effects [22]. Collective effects are aggregated group properties, and have previously been measured by the degree of unemployment, deprivation, distribution of economic sectors, housing quality, and percentage of the population with a certain income level [8, 11, 30, 35, 52, 53]. Contextual effects are related to a broader political, cultural and/or institutional context, for example the presence of infrastructure such as available health services [9, 52], but also ecological or environmental influences such as air pollution, noise pollution and temperature [40, 53].

In order to understand place effects on health, research should indeed consider higher-level variables too [41]. The impact of such effects seems to vary considerably in literature [1, 8, 18, 26, 43]. Research in the UK, Germany and the Netherlands points to a significant relation between geographic health differentiation and area deprivation (unemployment, illiteracy), availability of health-care facilities and aggregated lifestyle indicators (% of smokers and heavy drinkers) [17, 23, 39, 42]. Compared to area-level factors, individual health behaviours and social status seem to have become more influential over time however [24]. Some authors believe that area-level socio-economic characteristics play an intermediary role between individual SEP and health, since individuals with a low SEP are more common in socially deprived areas [53]. In such cases, the explanatory power of area-level characteristics is limited.

In Belgium, a few studies consider geographic mortality differences in relation to SEP, measured through home ownership status, housing comfort, household position and educational level [13, 14, 19]. These analyses show that individual SEP cannot fully explain observed geographic mortality patterns. Most of this research is based on data for the first half of the 1990s and does not consider area characteristics [13, 14].

This paper first wishes to describe the geographic pattern of all-cause mortality among middle-aged Belgian men during the more recent period 2001–2011. Second, we aim to probe into the role of individual and area-level socio-economic characteristics in explaining the observed geographic mortality differences.

## Methods

### Data

This study focuses on geographic differences in mortality among Belgian men aged 45 to 64. At these ages, socio-economic position (SEP) is relatively stable and easily measurable [12, 14]. In total 768,927 individuals are included in our analyses.

Data stem from a linkage between the Belgian 2001 census and follow-up register data on emigration and mortality during the period 2001–2011. Data are exhaustive and contain a rich set of socio-economic and socio-demographic variables at baseline.

### Indicators

The geographic unit for which we describe the mortality pattern is added as an explanatory variable, although conceptually we do not treat it as such. Descriptions are made both at the district level, Nomenclature of Territorial Units for Statistics level 3 (NUTS3) (*n* = 43), and the sub-district level (*n* = 68). The NUTS3-level constitutes the basis of the more detailed sub-district spatial division (Additional file 1: Figure S1). In NUTS3 units with a regional city, the district is split up into an urban and a non-urban part. If the regional city forms a city region [28], the urban agglomeration (central city plus densely built-up area around it) is taken as the urban part. Furthermore, some adjustments are made in order to keep a minimum of 50,000 and maximum of 550,000 residents within each spatial unit. This implies that some adjacent districts are grouped together, the central city and the rest of the urban agglomeration constitute two separate units in the large metropolitan cities of Brussels, Antwerp, Liège and Charleroi, and the Brussels-Capital Region is split up into an “inner-city” and “outer-city”. The only exception is the city of Arlon counting about 25,000 inhabitants only. The sub-district level is introduced into the analysis because NUTS3-level results are likely to hide much variation, whereas municipality-level results are not robust enough because of small numbers of deaths in some units [14]. Age is added as a control variable in all analyses.

In order to investigate the contribution of individual SEP to the geographic mortality pattern, several SEP indicators are introduced, and the resulting change in (sub-)district MRRs assessed. Educational level refers to the highest level obtained and consists of four categories corresponding to International Standard Classification of Education (ISCED) 2011 codes, namely no or lower education (ISCED 0–1), lower secondary education (ISCED 2), higher secondary education (ISCED 3–4) and higher education (ISCED 5–8). Occupational status is measured through three indicators: activity status, job category and activity sector. Activity status indicates whether someone is active on the labour market, job seeking, retired, unemployed for familial, social, personal, health-related or other reasons, or has never been active/not capable to answer/other. Someone’s job category can be civil servant or other employee, manager, self-employed or liberal profession, labourer, or other. Finally, the activity sector can be the primary, secondary, tertiary, quaternary, or another sector. Housing indicators are used as a proxy for lifetime economic affluence [3, 4, 22, 25] and consist of home ownership and the comfort level of the dwelling [51]. The effect of these housing indicators may be influenced by the impact of the household position: housing is not a purely individual SEP-indicator, but is rather influenced and shared by other members of the household. Household position is therefore introduced to the analyses as a control variable.

To explore the effect of area-level socio-economic characteristics on the mortality distribution in Belgium, several aggregated indicators often used in the literature [15] are included at the sub-district level: the percentage of individuals that completed higher education, the percentage of individuals that are employed, the percentages of labourers, the percentage of home owners, and the percentage of individuals living in a dwelling with the highest comfort level.

### Data analysis

Mortality rate ratios (MRR) are estimated through Poisson regression models in STATA 13. In order to establish the geographic mortality pattern, mortality is first fitted by district and controlled for age using single-level Poisson regression modelling. The same model is fitted by sub-district.

Second, we probe into the role of individual and area-level socio-economic characteristics. Each of the individual SEP indicators is added separately to the abovementioned single-level model to obtain an estimation of the crude effect of each of these indicators. Finally, a single-level multivariate model is run using district, individual SEP indicators, namely educational level, the three occupational status indicators, housing tenure and comfort level, and the control variables age and household position. These analyses are run at the sub-district level as well.

The impact of the individual SEP on the effect of (sub-)district is visible through the change in the (sub-)district MRRs after adjustment for individual SEP. Results will be shown by means of maps. The first map represents classes of MRRs controlled only for age, the second shows MRRs after adjustment for individual SEP controlled for household position in addition to age. The more the adjusted MRR approaches one after adjustment, the more likely it is that a mortality (dis-)advantage is partly attributable to the share of individuals with a certain SEP in this (sub-)district. Maps were constructed in QGIS, and MRR-classes are based on ‘natural breaks (jenks)’, aiming to find natural groupings of data to create classes. Caution is required when comparing maps before and after adjustment for individual SEP, since classes of MRRs used across maps are different. Therefore, color-codes do not represent the same values.

To explore the impact of area-level socio-economic characteristics, multilevel Poisson modelling is used in order to avoid problems of independence of research subjects within the same area [27]. Our data are divided into two levels: the individual level and the sub-district level. The GLAMM-program (general linear latent and mixed models) in STATA is used to fit random intercepts and random intercepts and slopes Poisson models. Random intercepts models allow the MRR to vary by sub-district, but assume the same effects from the independent variables across geographic units. We will illustrate this variability by sub-district with a table. A random intercepts and slopes model allows this effect from the independent variables on MRR to vary across sub-districts.

A random intercepts model with predictors at both levels is estimated by adding each aggregate indicator separately in a first stage, and a combination of significant variables in a second stage, of which we only keep the significant ones in this combined model. A table on this final model will be presented. The random intercepts and slopes models estimate the variability of individual SEP indicator effects between sub-districts. This allows us to explore whether effects of individual SEP-indicators on MRRs differ by sub-district, and will be shown in a table as well.

Schwarz’s Bayesian Information Criterion (BIC) is used to judge the goodness of fit of our models. This criterion takes the number of subjects and the number of parameters into account and helps to avoid overfitting [5].

## Results

### The geographic mortality pattern in Belgium

The Belgian mortality pattern by district is characterised by a distinct north–south pattern (Additional file 2: Figure S2 and Additional file 3: Table S1). The MRR of each district compared to the national average shows that nearly all of the northern (Flemish) districts have a significantly lower MRR compared to the Belgian average, namely lower than 1. This difference is not significant for Oostende, Aalst, Dendermonde and Oudenaarde. The opposite is true in the southern part of the country, premature mortality being higher in almost all Walloon districts, namely higher than 1. Nivelles is an exception, and the MRR for Arlon does not differ significantly from the Belgian average. The highest MRRs are observed in a strip from the southeast to the northwest, following the French border, the lowest MRRs in the northeast of the country. The MRR of the Brussels-Capital Region is comparable to that of a number of Walloon districts, for example Huy, Bastogne, Marche-en-Famenne and Namur.

**Fig. 1:**
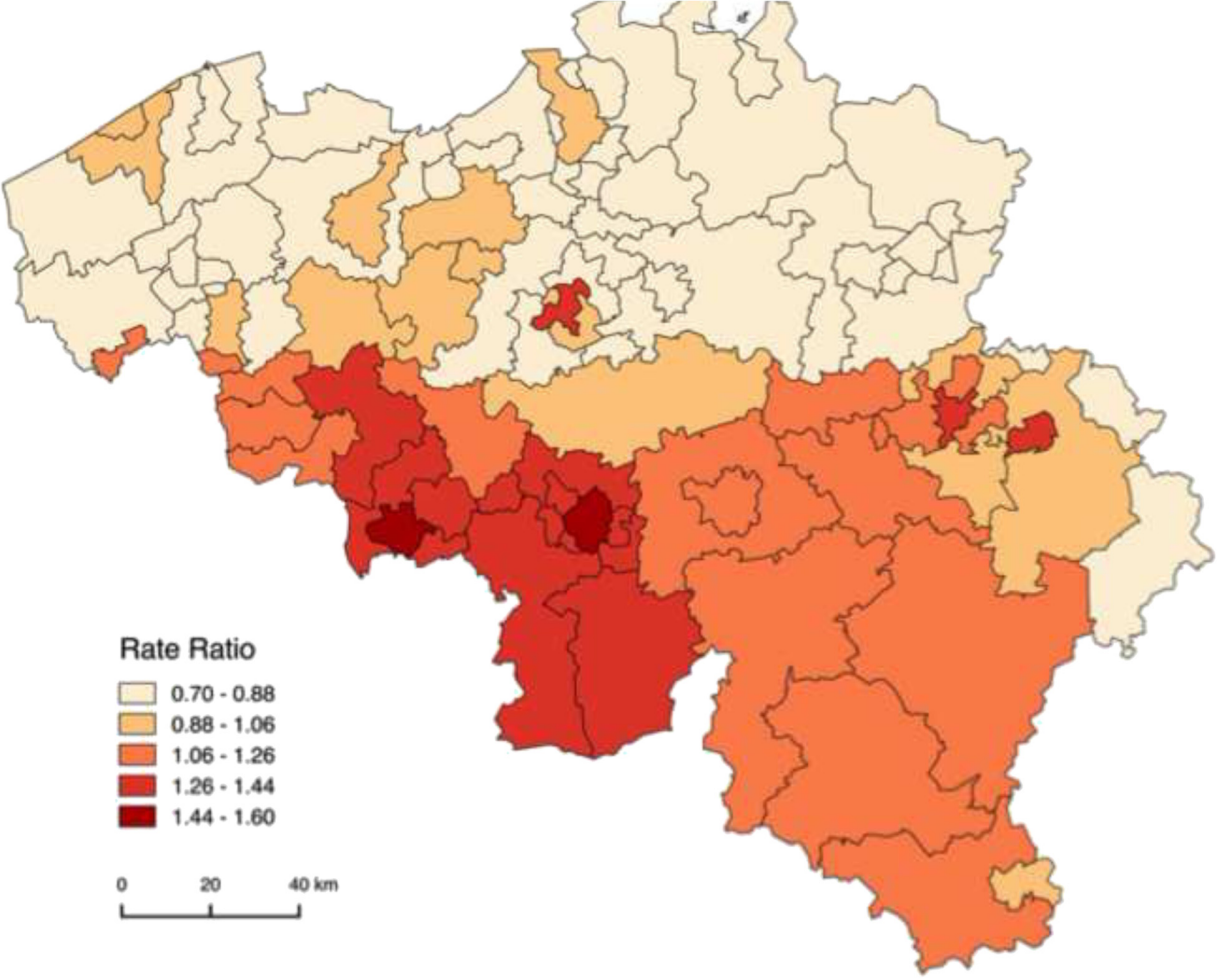
Geographic distribution of all-cause Mortality Rate Ratios (MRRs) by sub-district, controlled for age (Belgium, 2001–2011)

Figure 1 presents the age-adjusted geographic mortality distribution by sub-district. It confirms the north–south divide with very high MRRs in the Walloon province of Hainaut. The analysis by sub-district exemplifies the variation hidden within the NUTS3 districts. Especially urban areas within the Walloon region are characterised by high MRRs, particularly in Mons, Charleroi, Verviers and Liège. The higher Walloon MRRs are not significantly different from the Belgian average in the rural areas in Liège and Verviers. Nearly all Flemish sub-districts have lower MRRs than the Belgian average. For the city of Antwerp the lower MRR is not significant. A divide between a very high MRR for the inner city and a MRR just above the national mortality level for the outer city characterises the Brussels-Capital Region.

### Who you are: the effect of individual SEP on the geographic mortality pattern in Belgium

Crude models adjusting for one SEP-indicator at a time reveal that activity status leads to the largest changes in the district-level MRRs, followed by home ownership and housing comfort controlled for household position. In a multivariate model including educational level, the three indicators for occupational status, housing tenure and quality, as well as control variables age and household position, the SEP indicators generate the expected social gradient. In other words: a lower SEP characterized by a lower educational level, unemployment for a specific reason, a job as a labourer, social housing and lower housing comfort, results in significantly higher MRRs. Generally, this multivariate model does not change the observed mortality distribution at the district-level fundamentally, aside from increasing MRRs in the most advantaged districts and decreasing MRRs in the most disadvantaged districts (Additional file 3: Table S1 and Additional file 4: Figure S3). The multivariate model results in large decreases of the MRR in the Brussels-Capital Region (from 1.2 to 1.0) and in Oostende (1.0 to 0.9). Comparable decreases are observed in the Walloon region for the the districts of Charleroi (1.4 to 1.3), Mons (1.5 to 1.3) and Mouscron (1.1 to 1.0).

**Fig. 2:**
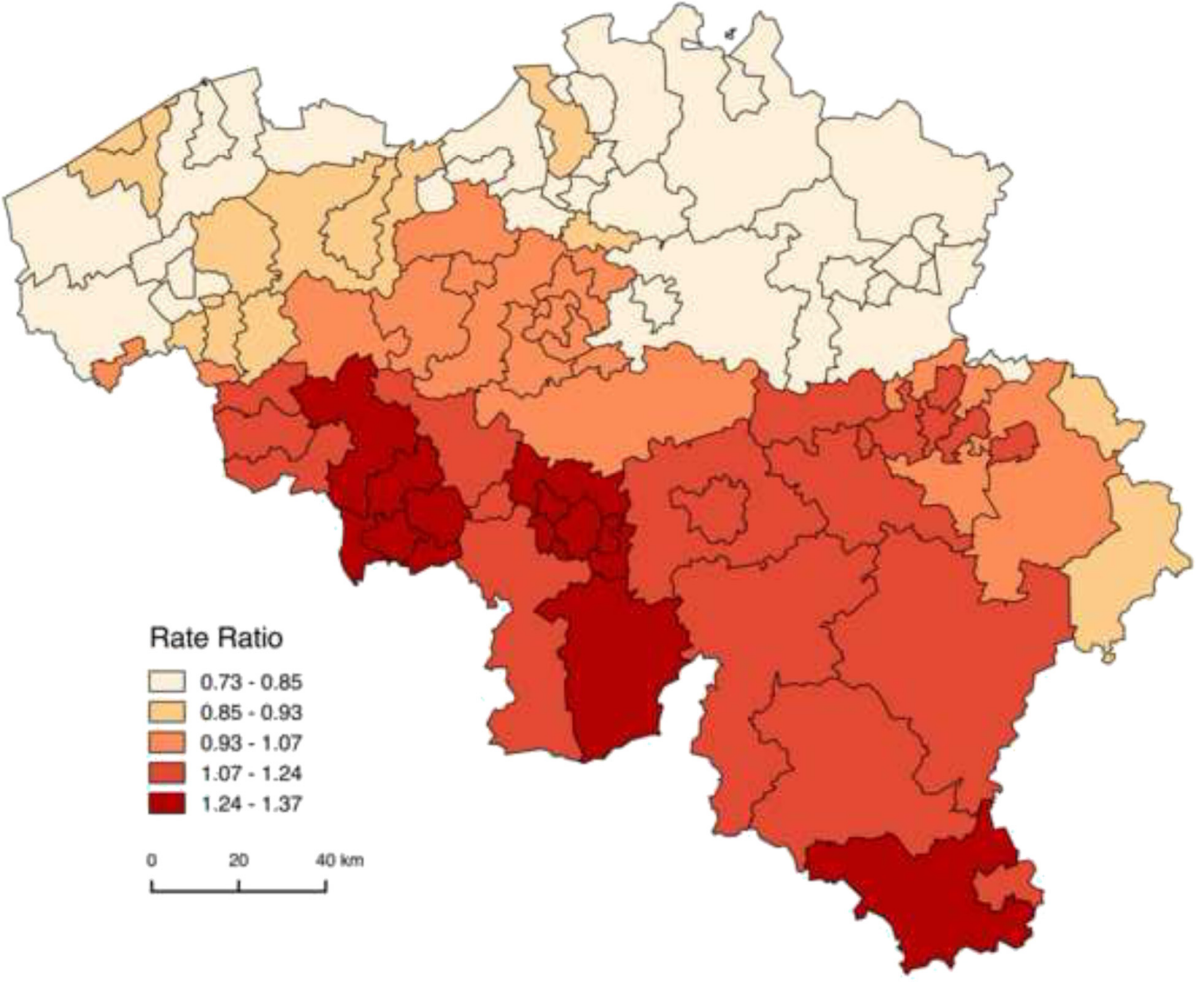
Geographic distribution of all-cause Mortality Rate Ratios (MRRs) by sub-district, controlled for age and household position, after adjustment for individual socio-economic position (Belgium, 2001–2011)

Adjusting for individual SEP causes slight changes in the geographic mortality pattern due to increases and decreases in MRRs for sub-districts as well. Changes occur with respect to the ranking of the most to the least disadvantaged sub-districts, but the overall observed sub-district mortality pattern does not radically differ (Additional file 5: Table S2). These sub-district analyses reveal that the decrease in MRR after adjustment for individual SEP for the Brussels-Capital Region at the district-level is largely due to a substantial decrease of the inner city MRR (1.3 to 1.0) after adjustment (Fig. 2). After this adjustment for individual SEP, the largest declines in MRR occur in the urban sub-districts of Charleroi and Mons and in urban Soignies, Liège and Verviers. Most decreases in MRR after adjustment visibly occur alongside the French border. On the other hand, the largest increases in MRR after adjustment are observed in the sub-districts of the province of Antwerp, in the central provinces Vlaams-Brabant and Brabant Wallon and in Arlon and Virton. In general, the increase in MRR after adjustment for individual SEP is most pronounced in rural areas surrounding the cities.

In Table 2, we show how the variability of the average MRR across sub-districts changed from a basic random intercepts model controlling for age (Model 1), over a random intercepts model controlling for age and household position, and adjusting for individual SEP (Model 2), to a random intercepts model taking both individual SEP and aggregate socio-economic characteristics of the sub-district into account (Model 3). Comparing Model 1 and 2, we see how the variability in the average MRR across sub-districts decreased due to adjustment for individual SEP. It appears to have an important effect on the geographic mortality distribution, but as the table shows: some variability remains that needs to be explained through different mechanisms.

### Where you live: the contribution of area-level socio-economic characteristics

The analyses combining individual and aggregate socio-economic characteristics at the sub-district level in multilevel random intercepts models with predictors at both levels reveal that the influence of aggregated socio-economic area characteristics on the mortality distribution beyond that of individual SEP is confined to three variables. The percentage employed, the percentage living in spacious and very comfortable housing and the percentage of labourers among middle-aged Belgian men have a small, but significant effect on the individual MRR. In a combined model with these three variables, the percentage of employed persons and labourers at the sub-district level remain of significant influence. Table 1 shows the MRRs for these two area-level indicators, as well as for individual SEP indicators in this model. This table shows that, regardless of the individual SEP, a 1 % increase in the proportion of employed individuals in a sub-district results in a 2 % decrease of the MRR for that sub-district. A 1 % increase in the proportion of labourers in a sub-district leads to a 1 % decrease of the sub-district mortality MRR.

**Table 1:** Mortality Rate Ratios (MRRs) and 95 % confidence intervals (CIs) in a random intercepts model with predictors at the individual and sub-district level, controlled for age and household position (Belgium, 2001–2011)

Variable	MRR (95 % CI)
Individual socio-economic position	
Educational level	
No/Lower education	1.00 (Ref.)
Lower secondary education	0.94 (0.92–0.96)***
Higher secondary education	0.91 (0.89–0.93)***
Higher education	0.75 (0.73–0.77)***
Employment status	
Active	1.00 (Ref.)
Job-seeking	1.66 (1.61–1.71)***
Retired	1.38 (1.35–1.41)***
Unemployed (specific reason)	2.67 (2.60–2.73)***
Never active/not capable of answer/other	2.28 (1.80–2.88)***
Job category	
Civil servant/other employee	1.00 (Ref.)
Manager	1.00 (0.96–1.05)
Self-employed/liberal profession	1.03 (1.01–1.06)*
Labourer	1.04 (1.02–1.07)***
Other	1.11 (1.06–1.15)***
Activity sector	
Primary	1.00 (Ref.)
Secondary	1.11 (1.06–1.17)***
Tertiary	1.18 (1.12–1.24)***
Quaternary	1.25 (1.19–1.32)***
Other	1.23 (1.16–1.30)***
Home ownership	
Owner/Usufructuary	1.00 (Ref.)
Tenant	1.37 (1.34–1.40)***
Tenant at a public institution	1.48 (1.44–1.53)***
Comfort level dwelling	
Insufficient comfort	1.00 (Ref.)
Basic comfort	0.93 (0.90–0.95)***
Good comfort	0.80 (0.77–0.82)***
Good comfort and spacious	0.79 (0.76–0.81)***
Very good comfort	0.72 (0.70–0.75)***
Area-level socio–economic characteristics	
% employed	0.98 (0.97–0.99)***
% labourers	0.99 (0.98–0.99)***
Variability intercept	0.01 (0.01–0.02)
BIC	547122.70

*p < 0.05; **p < 0.01; ***p < 0.001

Table 2 shows how the variability of the average MRR across sub-districts declined further when aggregated socio-economic characteristics of the sub-district are added to the analyses. Such higher-level indicators thus appear important in addition to individual SEP in explaining differing MRRs across sub-districts. Nevertheless, some variability remains, as observed through the Model 3 intercept variability.

**Table 2:** Variability in intercept, 95 % confidence intervals (CIs) and Schwarz’s Bayesian Information Criterion (BIC) for a random intercepts model controlling for age (Model 1), a random intercepts model controlling for age and household position while adjusting for individual socio–economic position (Model 2), and a random intercepts model adjusting for individual socio-economic position and aggregate socio-economic characteristics of the sub-district (Model 3) (Belgium, 2001–2011)

Model	Variability	95 % CI	BIC
Model 1	0.050	(0.035–0.070)	564510.69
Model 2	0.031	(0.022–0.044)	547144.41
Model 3	0.014	(0.010–0.020)	547135.77

Table 3 shows the variability in individual SEP-indicator effects across sub-districts, as observed through random intercepts and slopes models. These results reveal that there is some changeability in the effect of educational level, employment status, job category, activity sector, home ownership and comfort of the dwelling on MRRs across sub-districts. However, the table shows that the degree of difference across sub-districts is small, and goodness-of-fit shows that none of these models improve the random intercepts model with a combination of individual SEP and aggregate socio-economic characteristics.

**Table 3:** Variability in individual socio-economic position (SEP) indicator Mortality Rate Ratios (MRRs) with 95 % confidence intervals (CIs) and Schwarz’s Bayesian Information Criterion (BIC) in random intercepts and slopes models with predictors at both levels, controlled for age and household position (Belgium, 2001–2011)

SEP-indicator	Variability MRR (95 % CI)	BIC
Educational level	0.0127 (0.0099–0.01613)	547265.34
Employment status	0.0135 (0.0105–0.0172)	547219.84
Job category	0.0138 (0.0107–0.0177)	547196.08
Activity sector	0.0133 (0.0104–0.0170)	547268.04
Home ownership	0.0136 (0.0102–0.0180)	547135.40
Comfort level dwelling	0.0140 (0.0111–0.0177)	547255.52

## Discussion

This study describes the Belgian geographic all-cause mortality pattern in middle-aged men, and reveals to what extent this pattern can be attributed to individual and area socio-economic characteristics. Individual SEP contributes to the geographic mortality pattern in Belgium, but cannot explain all of the observed variability in MRRs across geographic units. Two aggregated area-level variables, the percentage of employed men and the percentage of labourers in a sub-district, have a statistically significant protective effect on the MRRs regardless of individual SEP.

In this study, results from single-level Poisson regression models reconfirm the classic north–south divide in Belgian mortality levels, as well as the high mortality zone stretching from the southeast to the northwest of the country that have previously been observed [13, 14, 37, 38].

The finding that individual SEP indicators contribute to the geographic mortality pattern in 2001–2011 is consistent with international research results [7, 9, 10, 35]. For some areas a distribution of low individual SEP seems to result in a mortality disadvantage, observed through high MRRs that decline steeply after adjustment for individual SEP-indicators, for example for urban areas in Wallonia, Brussels, and Antwerp. For other areas, we observe a mortality advantage, likely due to distribution of high SEP, observed through low MRRs that increase after adjustment, like for most of the northern sub-districts, Arlon and Virton. The impact of individual SEP in Belgium was previously calculated at the district-level for the period 1991–1995, generally leading to the same conclusion for this earlier period [13, 14].

In this paper we examined additional effects on MRR of aggregated socio-economic factors at the sub-district level in random intercepts Poisson regression models with predictors at the individual and sub-district level. Evidence from neighbouring countries underlines the role of area deprivation, social conditions and aggregated lifestyle indicators [17, 23, 39, 42]. Our Belgian results show an effect of aggregated area-level socio-economic characteristics added to that of individual SEP. Two indicators play a significant role: the percentage of employed middle-aged men and the percentage of labourers in a sub-district. An increase in these indicators is associated with a decrease in the individual MRR, regardless of the personal SEP. German research observes a smaller role of area-level characteristics as opposed to individual SEP [24, 53]. These authors assign socio-economic characteristics of the area an intermediary role between individual SEP and health, as individuals with a low SEP are more common in socially deprived areas to begin with. Nevertheless, a review by Meijer et al. [33] assigns an additional role to the area socio-economic characteristics: the concentration of individuals with high SEP will likely attract more health promoting provisions such as sports clubs, healthy food stores, etc., because they have easier access to them. Also, the social environment will be affected in terms of safety, social cohesion, local institutions and norms. All individuals, regardless of SEP, could benefit from that. Such an effect over that of individual SEP distribution appears to be present in our results as well.

Due to the fact that some sub-district MRRs increased and others decreased when we added individual SEP-indicators to the single-level models, we explored whether the effect of those SEP indicators varies substantially across sub-districts. Our study results reveal that there is in fact some variability in the effect of individual SEP indicators across sub-districts, but only to a limited extent.

After adjustment for both individual SEP and aggregate socio-economic variables at the level of the sub-district, we are left with an important geographic variation in MRRs that remains unexplained. Remaining differences may be due to geographic variation in health behaviour independent of SEP, health service provision, environmental pollution, and differences in infrastructure. A first limitation of this study is therefore that we only use aggregate socio-economic area-level variables to go beyond the effects of individual SEP, as we focused only on socio-economic contributions to mortality.

Environmental exposures and care-related characteristics of the sub-districts may have an added value [9, 40, 52, 53]. Future research using this database my include a number variables related to individuals’ perceptions of the neighbourhood, for example air pollution, presence of green spaces, health care facilities, etc. Alternatively, other databases with objective measurements on these topics could be used.

Renard et al. [38] and colleagues showed the importance of varying health behaviour and population density, for example, through alcohol consumption in the north of France that may be crossing the border to Belgium, leading to higher alcohol-related mortality in border-areas from the south-east to the north-west of the country. Also, long distances travelled by cars on fast roads in the more rural, less densely populated, Walloon region is linked to more road traffic accidents. Information on such aspects is not collected through the census and can therefore not directly be included in our analyses. No Belgian study has managed to combine socio-economic variables, mortality, health behaviour data (from the Health Interview Survey, for example), information about health care provision, and data on environmental exposures in one model so far. Additionally, socio-cultural and historical information on Belgian areas would likely contribute to our understanding of the observed mortality distribution.

Spatial correlation between bordering areas was not taken into account in this paper. Finally, conclusions of this paper cannot be generalised to younger or older age groups, women or foreign nationals living in Belgium due to our focus on Belgian middle-aged men. Regardless, different mechanisms are to be taken into account when including women and persons with a foreign nationality, such as larger work-family conflicts for women and different prior exposures for non-Belgians [14, 34]. Although some of these mechanisms might also apply to our analysis of middle-aged Belgian men to some extent due to, for example, naturalisation of migrants, including women and non-Belgians would have partly necessitated a shift in focus of this study.

This paper contributes to the body of evidence on geographic mortality distributions. It gives an update of analyses previously performed in the nineties on the Belgian geographic all-cause mortality pattern and on the role of individual SEP in explaining geographic mortality differences. Moreover, it adds a number of previously unexplored issues. Firstly, comparable analyses of all-cause mortality were never performed at the sub-district level. Previous publications focused on the district level. The sub-district division allows for a more nuanced picture, since these units allow us to distinguish between mortality levels in urban and rural areas. Scrutinizing area-level effects using multilevel modelling is also a novel contribution to the body of evidence. Undoubtedly, additional strengths of this paper are the use of an exhaustive dataset containing a large variety of socio-economic and socio-demographic variables. Finally, the exhaustiveness of the linked census-register dataset avoids a numerator-denominator bias: results are unlikely to be over- or underestimated because data on both the deaths and the population at risk stem from the same source.

## Conclusions

This paper maps out the geographic all-cause mortality pattern in Belgian men aged 45–64 for the period 2001–2011, reconfirming a clear north–south divide with a disadvantaged position for the Brussels-Capital Region and a high all-cause mortality zone stretching from the southeast to the northwest of the country. For a number of areas, the distribution of a rather advantaged or disadvantaged SEP of inhabitants contributes to the observed MRRs. The added effect of area-level socio-economic characteristics in this equation is important, but not sufficient in explaining the Belgian mortality distribution for middle-aged men. Our results show that the complex process leading up to geographical mortality differences is determined by more than individual SEP and area-level socio-economic characteristics. Further research might attempt to include information on health behaviour, socio-cultural and environmental factors, indicators on health care, and on the historical context in order to further explain the observed mortality pattern in Belgium.

Findings identify areas for which SEP-oriented or SEP–specific public health measures might be warranted in the short term in order to decrease the observed mortality disadvantage to some extent. In the long term, however, interventions aiming to prevent an unequal distribution of SEP are more likely to contribute to gains in health and mortality.

### Ethical approval

Using the abovementioned data for research and publication has been approved by the Privacy Commission. This study as well as the data adhere to the ethical code of scientific research in Belgium.

## Additional files

Additional file 1: Figure S1. Map of Belgian sub-districts. (PNG 519 kb) Additional file 2: Figure S2. Geographic distribution of all-cause Mortality Rate Ratios (MRRs) by district, controlled for age (Belgium, 2001–2011). (PNG 639 kb) Additional file 3: Table S1. All-cause Mortality Rate Ratios (MRRs) and 95 % confidence intervals (CIs) by district controlled for age, before and after adjustment for individual socio-economic position and household position (Belgium, 2001–2011). (DOCX 118 kb) Additional file 4: Figure S3. Geographic distribution of all-cause Mortality Rate Ratios (MRRs) by district, controlled for age and household position, after adjustment for individual socio-economic position (Belgium, 2001–2011). (PNG 649 kb) Additional file 5: Table S2. All-cause Mortality Rate Ratios (MRRs) and 95 % confidence intervals (CIs) in random intercepts models with predictors at the individual and sub-district level, controlled for age and household position, ranked by descending Mortality Rate Ratio (Belgium, 2001–2011). (DOCX 152 kb)

## References

[1] Anderson R, Sorlie P, Backlund E (1997). Mortality effects of community socioeconomic status. Epidemiology.

[2] Avendano M, Kunst a E, Huisman M, Lenthe FV, Bopp M, Regidor E, Mackenbach, JP. Socioeconomic status and ischaemic heart disease mortality in 10 western European populations during the 1990s. Heart. 2006;92(4):461–7. http://doi.org/10.1136/hrt.2005.065532. 10.1136/hrt.2005.065532 PMC1860902 16216862

[3] Bang D, Manemann S, Gerber Y, Roger V, Lohse C, Rand-Weaver J, Juhn Y. A Novel Socioeconomic Measure Using Individual Housing Data in Cardiovascular Outcome Research. Int J Environ Res Public Health. 2014;11(11):11597–615. http://doi.org/10.3390/ijerph111111597. 10.3390/ijerph111111597 PMC4245632 25396769

[4] Blázquez M, Cottini E, Herrarte A (2013). The socioeconomic gradient in health: how important is material deprivation?. J Econ Inequal.

[5] Buckland ST, Burnham KP, Augustin NH (1997). Model Selection: An Integral Part of Inference. Biometrics.

[6] Diez Roux AV (2002). A glossary for multilevel analysis. J Epidemiol Community Health.

[7] Ecob R, Jones K (1998). Mortality variations in England and Wales between types of place: an analysis of the ONS longitudinal study. Soc Sci Med.

[8] Elstad JI (2011). Does the socioeconomic context explain both mortality and income inequality? Prospective register-based study of Norwegian regions. Int J Equity Health.

[9] Fang P, Dong S, Xiao J, Liu C, Feng X, Wang Y (2010). Regional inequality in health and its determinants: evidence from China. Health Policy.

[10] Fernandez-Martinez B, Prieto-Flores M-E, Forjaz MJ, Fernández-Mayoralas G, Rojo-Pérez F, Martínez-Martín P (2012). Self-perceived health status in older adults: regional and sociodemographic inequalities in Spain. Rev Saude Publica.

[11] Franzini L, Giannoni M (2010). Determinants of health disparities between Italian regions. BMC Public Health.

[12] Gadeyne S (2006). The Ultimate Inequality: Socio-economic differences in all-cause and cause-specific Mortality in Belgium in the First Part of the 1990s.

[13] Gadeyne S, Deboosere P (2002). Can regional patterns of mortality in Belgium be explained by individual socio-economic characteristics?. Reflets et perspectives de la vie économique, Tome XLI.

[14] Gadeyne S, Deboosere P (2002). Socio-economische ongelijkheden in sterfte op middelbare leeftijd in België.

[15] Galobardes B, Lynch J, Smith GD (2007). Measuring socioeconomic position in health research. Br Med Bull.

[16] Galobardes B, Shaw M, Lawlor DA, Lynch JW, Davey Smith G (2006). Indicators of socioeconomic position (part 2). J Epidemiol Community Health.

[17] Groenewegen PP, Westert GP, Boshuizen HC (2003). Regional differences in healthy life expectancy in the Netherlands. Public Health.

[18] Haan M, Kaplan GA, Camacho T (1987). Poverty and health. Prospective evidence from the Almeda County study. Am J Epidemiol.

[19] Hagedoorn P, Vandenheede H, Willaert D, Vanthomme K, Gadeyne S (2016). Regional Inequalities in Lung Cancer Mortality in Belgium at the Beginning of the 21st Century : The Contribution of Individual and Area-Level Socioeconomic Status and Industrial Exposure. PLoS One.

[20] Huie SAB (2001). The Concept of Neighborhood in Health and Mortality Research. Sociol Spectr.

[21] Huisman M, Kunst AE, Andersen O, Bopp M, Borgan J, Borrell C, Costa G, Deboosere P, Desplanques G, Donkin A, Gadeyne S, Minder C, Regidor E, Spadea T, Valkonen T, Mackenbach JP (2004). Socioeconomic inequalities in mortality among elderly people in 11 European populations. J Epidemiol Community Health.

[22] Kawachi I, Subramanian SV, Almeida-Filho N (2002). A glossary for health inequalities. J Epidemiol Community Health.

[23] Kibele EUB, Klüsener S, Scholz RD (2015). Regional Mortality Disparities in Germany: Long-Term Dynamics and Possible Determinants. Kolner Zeitschrift Fur Soziologie Und Sozialpsychologie.

[24] Kuhn J, Zirngibl A, Wildner M, Caselmann WH, Kerscher G (2006). Regional mortality differences in Bavaria. Gesundheitswesen.

[25] Laaksonen M, Tarkiainen L, Martikainen P (2009). Housing wealth and mortality: A register linkage study of the Finnish population. Soc Sci Med.

[26] LeClere FB, Rogers RG, Peters KD (1997). Ethnicity and Mortality in the United States: Individual and Community Correlates. Soc Forces.

[27] Luke DA (2004). Multilevel modeling.

[28] Luyten S, Van Hecke E (2007). De Belgische stadsgewesten 2001.

[29] Macintyre S (1997). The Black Report and beyond: what are the issues?. Soc Sci Med.

[30] Mangano A (2010). An analysis of the regional differences in health care utilization in Italy. Health Place.

[31] Martelin T (1994). Mortality by indicators of socioeconomic status among the Finnish elderly. Soc Sci Med.

[32] Matthews FE, Miller LL, Brayne C, Jagger C (2006). Regional differences in multidimensional aspects of health: findings from the MRC cognitive function and ageing study. BMC Public Health.

[33] Meijer M, Röhl J, Bloomfield K, Grittner U (2012). Do neighborhoods affect individual mortality? A systematic review and meta-analysis of multilevel studies. Soc Sci Med.

[34] Mishra GD, Ball K, Dobson AJ, Byles JE, Warner-Smith P (2001). The measurement of socio-economic status: investigation of gender-and age-specific indicators in Australia: National Health Survey 1995. Soc Indic Res.

[35] Pirani E, Salvini S (2011). Socioeconomic Inequalities and Self-Rated Health: A Multilevel Study of Italian Elderly. Popul Res Policy Rev.

[36] Raes V, Kerkhofs E, Louckx F (1993). Sociale ongelijkheid en verschillen in gezondheid.

[37] Renard F, Tafforeau J, Deboosere P (2014). Premature mortality in Belgium in 1993–2009: leading causes, regional disparities and 15 years change. Arch Public Health.

[38] Renard F, Tafforeau J, Deboosere P (2015). Mapping the cause-specific premature mortality reveals large between-districts disparity in Belgium, 2003–2009. Arch Public Health.

[39] Riva M, Curtis S, Gauvin L, Fagg J (2009). Unravelling the extent of inequalities in health across urban and rural areas: Evidence from a national sample in England. Soc Sci Med.

[40] Romero-Lankao P, Qin H, Borbor-Cordova M (2013). Exploration of health risks related to air pollution and temperature in three Latin American cities. Soc Sci Med.

[41] Schwartz SH (1994). The fallacy of the ecological fallacy: The potential misuse of a concept and the consequences. Am J Public Health.

[42] Senior M, Williams H, Higgs G (2000). Urban–rural mortality differentials: controlling for material deprivation. Soc Sci Med.

[43] Smith GD, Hart C, Watt G, Hole D, Hawthorne V (1998). Individual social class, area-based deprivation, cardiovascular disease risk factors, and mortality: the Renfrew and Paisley Study. J Epidemiol Community Health.

[44] Stronks K, van de Mheen D, Mackenbach JP (1993). Achtergronden van sociaal-economische gezondheidsverschillen. Een overzicht van de literatuur en een onderzoeksmodel. Tijdschrift Voor Sociale Gezondheidszorg.

[45] Sun S, Chen J, Johannesson M, Kind P, Xu L, Zhang Y, Burström K. Regional differences in health status in China: population health-related quality of life results from the National Health Services Survey 2008. Health Place. 2011;17(2):671–80. http://doi.org/10.1016/j.healthplace.2011.01.007. 10.1016/j.healthplace.2011.01.007 21334961

[46] Tunstall HVZ, Shaw M, Dorling D (2004). Places and health. J Epidemiol Community Health.

[47] Van der Heyden JH, Schaap MM, Kunst AE, Esnaola S, Borrell C, Cox B, Van Oyen H. Socioeconomic inequalities in lung cancer mortality in 16 European populations. Lung Cancer. 2009;63(3):322–30. http://dx.doi.org/10.1016/j.lungcan.2008.06.006. 10.1016/j.lungcan.2008.06.006 18656277

[48] Van Oyen H, Bossuyt N, Deboosere P, Gadeyne S, Abatih E, Demarest SS (2005). Differential inequity in health expectancy by region in Belgium. Sozial- Und Präventivmedizin.

[49] Van Oyen H, Bossuyt N, Deboosere P, Gadeyne S, Tafforeau J (2002). Differences in health expectancy indicators in Belgium by region. Archives of Public Health.

[50] Van Oyen H, Tafforeau J, Roelands M (1996). Regional inequities in health expectancy in Belgium. Soc Sci Med.

[51] Vanneste, D., Thomas, I., & Goossens, L. (2007). Woning en woonomgeving in België. Monografieën van de Sociaal-Economische Enquête 2001, nummer 2.

[52] Voigtländer S, Berger U, Razum O (2010). Increasing regional disparities in living conditions in Germany and their role in the explanation of health inequalities. Gesundheitswesen (Bundesverband Der Ärzte Des Öffentlichen Gesundheitsdienstes (Germany)).

[53] Voigtländer S, Berger U, Razum O (2010). The impact of regional and neighbourhood deprivation on physical health in Germany: a multilevel study. BMC Public Health.

[54] WHO (2007). The World Health Organization on Health Inequality, Inequity, and Social Determinants of Health. Popul Dev Rev.

