# Supplementary figures and images for: The geographic pattern of Belgian mortality: can socio-economic characteristics explain area differences?
Source: Arch Public Health. 2016 Jun 8;74:22. doi: 10.1186/s13690-016-0135-y (PMC4897960; doi:10.1186/s13690-016-0135-y)

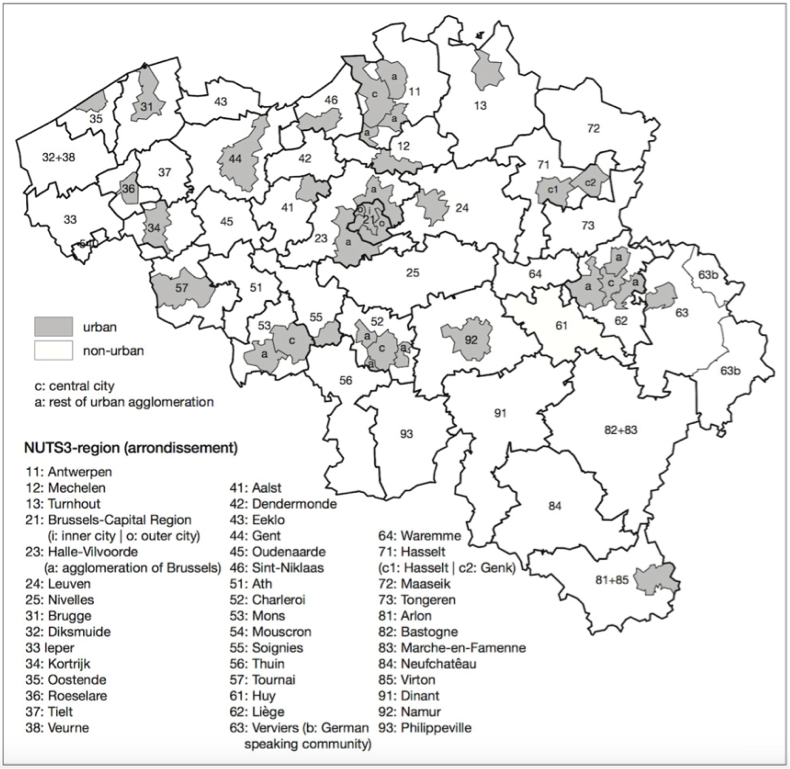

Supplement: Additional file 1: Figure S1. — Map of Belgian sub-districts. (PNG 519 kb) [file 13690_2016_135_MOESM1_ESM.png]

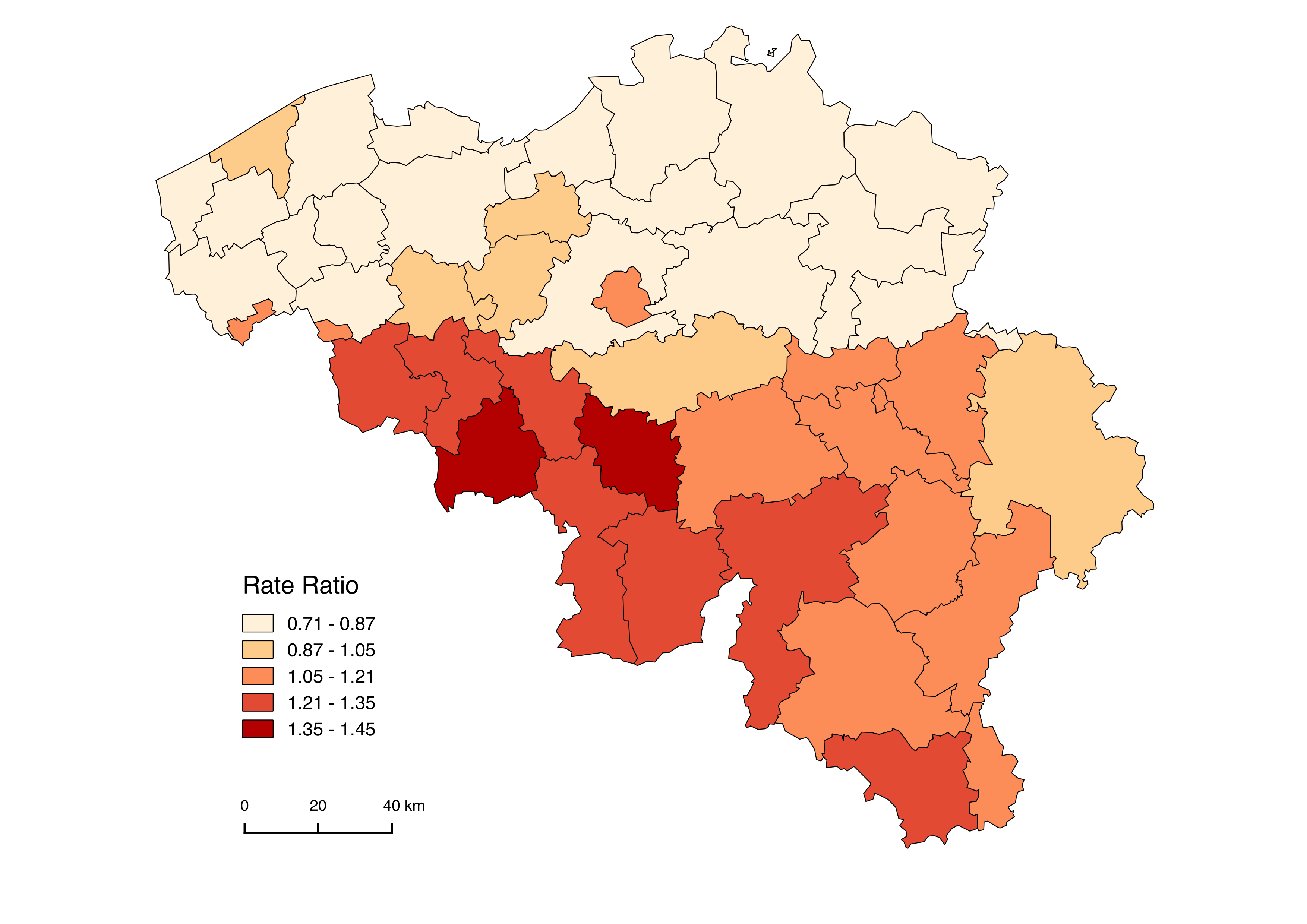

Supplement: Additional file 2: Figure S2. — Geographic distribution of all-cause Mortality Rate Ratios (MRRs) by district, controlled for age (Belgium, 2001–2011). (PNG 639 kb) [file 13690_2016_135_MOESM2_ESM.png]

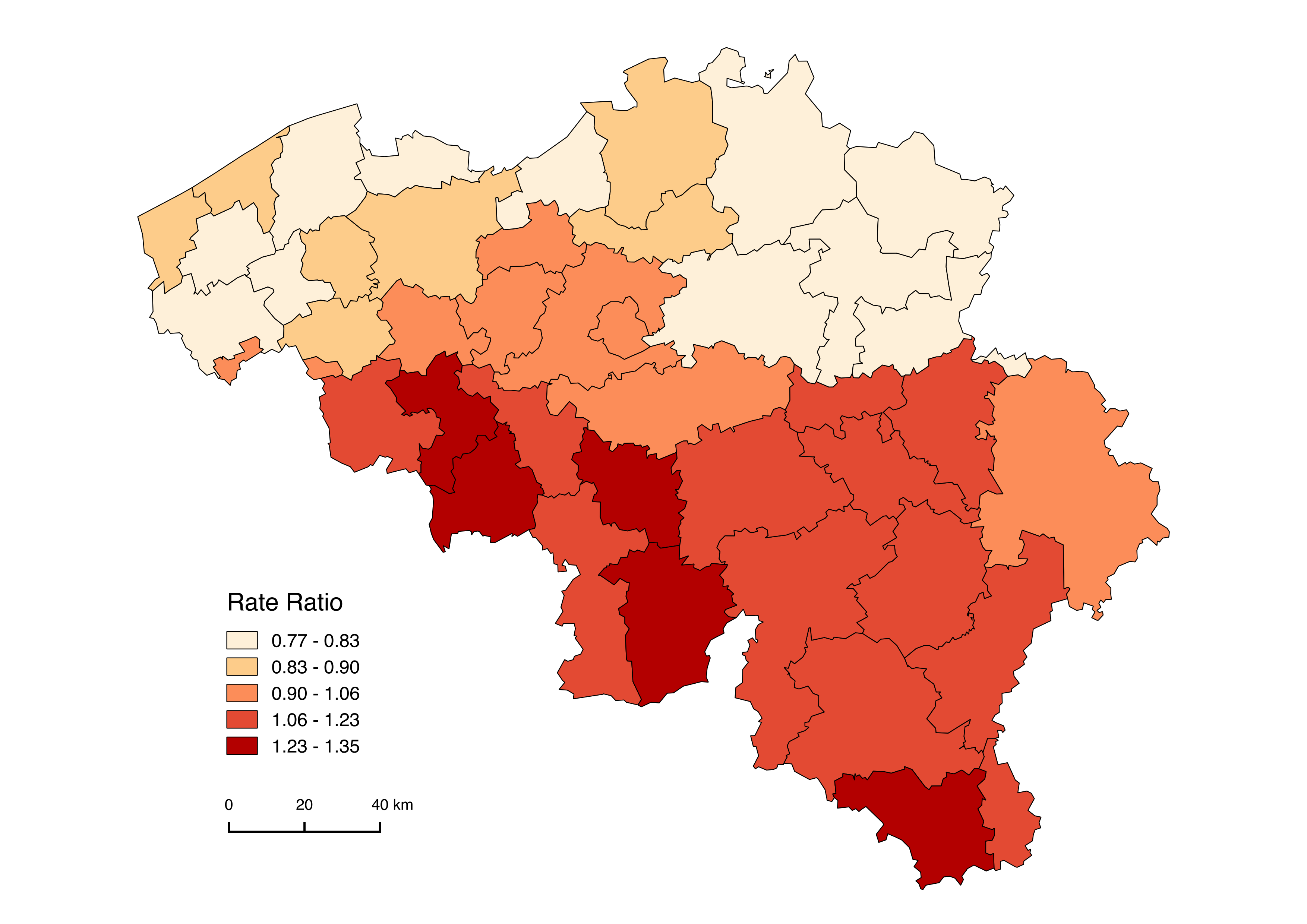

Supplement: Additional file 4: Figure S3. — Geographic distribution of all-cause Mortality Rate Ratios (MRRs) by district, controlled for age and household position, after adjustment for individual socio-economic position (Belgium, 2001–2011). (PNG 649 kb) [file 13690_2016_135_MOESM4_ESM.png]
