# Supplementary material for: The geographic pattern of Belgian mortality: can socio-economic characteristics explain area differences?
Source: Arch Public Health. 2016 Jun 8;74:22. doi: 10.1186/s13690-016-0135-y (PMC4897960; doi:10.1186/s13690-016-0135-y)
Supplement: Additional file 3: Table S1. — All-cause Mortality Rate Ratios (MRRs) and 95 % confidence intervals (CIs) by district controlled for age, before and after adjustment for individual socio-economic position and household position (Belgium, 2001–2011). (DOCX 118 kb) [file 13690_2016_135_MOESM3_ESM.docx]

***Table S1.*** *All-cause MRRs and 95% confidence intervals by district controlled for age, before and after adjustment for individual SEP and household position with 95% confidence intervals (2001-2011)*

| **District** | **Basic model** | | **Multivariate model** | |
| --- | --- | --- | --- | --- |
| Antwerp | 0.82 | [0.80 - 0.84] | 0.85 | [0.83 - 0.87] |
| Mechelen | 0.82 | [0.79 - 0.86] | 0.84 | [0.81 - 0.88] |
| Turnhout | 0.73 | [0.70 - 0.76] | 0.77 | [0.74 - 0.80] |
| Brussels Capital | 1.16 | [1.13 - 1.20] | 1.03 | [0.99 - 1.06] |
| Halle-Vilvoorde | 0.86 | [0.83 - 0.89] | 0.96 | [0.93 - 0.99] |
| Leuven | 0.77 | [0.74 - 0.79] | 0.83 | [0.80 - 0.86] |
| Nivelles | 0.92 | [0.89 - 0.96] | 1.04 | [1.00 - 1.09] |
| Bruges | 0.80 | [0.76 - 0.84] | 0.82 | [0.78 - 0.85] |
| Diksmuide | 0.87 | [0.78 - 0.97] | 0.82 | [0.74 - 0.92] |
| Ieper | 0.84 | [0.77 - 0.90] | 0.82 | [0.76 - 0.88] |
| Kortrijk | 0.85 | [0.81 - 0.89] | 0.89 | [0.85 - 0.93] |
| Oostende | 0.98 | [0.92 - 1.03] | 0.90 | [0.85 - 0.95] |
| Roeselare | 0.82 | [0.77 - 0.87] | 0.82 | [0.77 - 0.88] |
| Tielt | 0.82 | [0.76 - 0.89] | 0.86 | [0.79 - 0.93] |
| Veurne | 0.87 | [0.79 - 0.95] | 0.84 | [0.76 - 0.92] |
| Aalst | 0.98 | [0.93 - 1.02] | 0.99 | [0.94 - 1.03] |
| Dendermonde | 0.96 | [0.91 - 1.01] | 0.95 | [0.91 -1.00 ] |
| Eeklo | 0.83 | [0.76 - 0.90] | 0.81 | [0.74 - 0.88] |
| Ghent | 0.85 | [0.83 - 0.89] | 0.88 | [0.85 - 0.91] |
| Oudenaarde | 0.95 | [0.89 - 1.01] | 0.98 | [0.91 - 1.04] |
| Sint-Niklaas | 0.81 | [0.77 - 0.85] | 0.83 | [0.78 - 0.87] |
| Ath | 1.35 | [1.26 - 1.44] | 1.28 | [1.20 - 1.38] |
| Charleroi | 1.43 | [1.38 - 1.48] | 1.27 | [1.23 - 1.32] |
| Mons | 1.45 | [1.39 - 1.52] | 1.33 | [1.27 - 1.39] |
| Mouscron | 1.13 | [1.03 - 1.24] | 1.05 | [0.96 - 1.15] |
| Soignies | 1.27 | [1.20 - 1.34] | 1.20 | [1.14 - 1.27] |
| Thuin | 1.31 | [1.24 - 1.38] | 1.23 | [1.16 - 1.30] |
| Tournai | 1.23 | [1.16 - 1.30] | 1.14 | [1.08 - 1.21] |
| Huy | 1.18 | [1.10 - 1.26] | 1.19 | [1.12 - 1.27] |
| Liège | 1.21 | [1.17 - 1.25] | 1.13 | [1.10 - 1.17] |
| Verviers | 1.05 | [1.00 - 1.09] | 1.06 | [1.01 - 1.11] |
| Waremme | 1.14 | [1.06 - 1.24] | 1.21 | [1.12 - 1.31] |
| Hasselt | 0.76 | [0.73 - 0.79] | 0.80 | [0.76 - 0.83] |
| Maaseik | 0.71 | [0.67 - 0.76] | 0.77 | [0.73 - 0.82] |
| Tongeren | 0.79 | [0.75 - 0.84] | 0.82 | [0.78 - 0.87] |
| Arlon | 1.08 | [0.97 - 1.19] | 1.18 | [1.06 - 1.30] |
| Bastogne | 1.16 | [1.04 - 1.29] | 1.17 | [1.05 - 1.31] |
| Marche-en-Famenne | 1.19 | [1.08 - 1.30] | 1.15 | [1.04 - 1.26] |
| Neufchâteau | 1.20 | [1.10 - 1.31] | 1.21 | [1.11 - 1.32] |
| Virton | 1.27 | [1.15 - 1.40] | 1.35 | [1.22 - 1.48] |
| Dinant | 1.25 | [1.17 - 1.33] | 1.20 | [1.13 - 1.28] |
| Namur | 1.15 | [1.10 - 1.20] | 1.15 | [1.11 - 1.20] |
| Philippeville | 1.32 | [1.22 - 1.43] | 1.30 | [1.19 - 1.40] |
| **BIC** | 565132.7283 | | 547452.0071 | |
| **Pseudo-R2** | 0.0313 | | 0.0622 | |
