# Supplementary material for: The geographic pattern of Belgian mortality: can socio-economic characteristics explain area differences?
Source: Arch Public Health. 2016 Jun 8;74:22. doi: 10.1186/s13690-016-0135-y (PMC4897960; doi:10.1186/s13690-016-0135-y)
Supplement: Additional file 5: Table S2. — All-cause Mortality Rate Ratios (MRRs) and 95 % confidence intervals (CIs) in random intercepts models with predictors at the individual and sub-district level, controlled for age and household position, ranked by descending Mortality Rate Ratio (Belgium, 2001–2011). (DOCX 152 kb) [file 13690_2016_135_MOESM5_ESM.docx]

***Table S2.*** *MRRs and 95% confidence intervals in random intercepts models with predictors at the individual and sub-district level, controlled for age and household position, ranked by descending MRR (2001-2011)*

| **Sub-district** | **Basic model** | | | **Multivariate model** | | |
| --- | --- | --- | --- | --- | --- | --- |
| Mons-urban agglomeration | 1.60 | [1.49 - 1.71] | 1 | 1.37 | [1.28 - 1.47] | 1 |
| Charleroi-city | 1.55 | [1.47 - 1.63] | 2 | 1.30 | [1.24 - 1.37] | 6 |
| Liège-city | 1.44 | [1.37 - 1.52] | 3 | 1.19 | [1.13 - 1.26] | 17 |
| Mons-city | 1.42 | [1.32 - 1.53] | 4 | 1.31 | [1.22 - 1.41] | 4 |
| Charleroi-urban agglomeration | 1.39 | [1.28 - 1.50] | 5 | 1.26 | [1.17 - 1.36] | 9 |
| Soignies-city | 1.36 | [1.24 - 1.49] | 6 | 1.18 | [1.07 - 1.29] | 19 |
| Ath | 1.36 | [1.26 - 1.46] | 7 | 1.30 | [1.21 - 1.39] | 7 |
| Mons-other | 1.35 | [1.24 - 1.47] | 8 | 1.34 | [1.23 - 1.46] | 2 |
| Philippeville | 1.33 | [1.23 - 1.45] | 9 | 1.31 | [1.21 - 1.42] | 5 |
| Charleroi-other | 1.33 | [1.25 - 1.41] | 10 | 1.28 | [1.20 - 1.36] | 8 |
| Thuin | 1.32 | [1.25 - 1.39] | 11 | 1.24 | [1.18 - 1.32] | 10 |
| Brussel-inner city | 1.30 | [1.25 - 1.36] | 12 | 1.04 | [1.00 - 1.08] | 31 |
| Verviers-city | 1.30 | [1.20 - 1.40] | 13 | 1.21 | [1.12 - 1.30] | 15 |
| Dinant | 1.26 | [1.18 - 1.34] | 14 | 1.22 | [1.14 - 1.30] | 14 |
| Tournai-other | 1.26 | [1.16 - 1.36] | 15 | 1.18 | [1.08 - 1.28] | 18 |
| Soignies-other | 1.24 | [1.16 - 1.32] | 16 | 1.23 | [1.16 - 1.32] | 11 |
| Arlon-other | 1.23 | [1.14 - 1.34] | 17 | 1.32 | [1.22 - 1.43] | 3 |
| Liège-urban agglomeration | 1.22 | [1.17 - 1.28] | 18 | 1.17 | [1.12 - 1.22] | 21 |
| Tournai-city | 1.21 | [1.12 - 1.32] | 19 | 1.14 | [1.05 - 1.23] | 24 |
| Neufchâteau | 1.21 | [1.11 - 1.32] | 20 | 1.22 | [1.12 - 1.34] | 13 |
| Namur-city | 1.19 | [1.12 - 1.27] | 21 | 1.17 | [1.10 - 1.25] | 20 |
| Huy | 1.19 | [1.11 - 1.27] | 22 | 1.20 | [1.13 - 1.29] | 16 |
| Bastogne - March-en-Famenne | 1.18 | [1.1 - 1.23] | 23 | 1.17 | [1.09 - 1.26] | 22 |
| Waremme | 1.15 | [1.07 - 1.25] | 24 | 1.22 | [1.13 - 1.32] | 12 |
| Namur-other | 1.14 | [1.08 - 1.20] | 25 | 1.16 | [1.10 - 1.22] | 23 |
| Mouscron | 1.14 | [1.04 - 1.25] | 26 | 1.06 | [0.97 - 1.16] | 27 |
| Brussels-outer city | 1.06 | [1.02 - 1.11] | 27 | 1.04 | [1.00 - 1.08] | 30 |
| Aalst-city | 1.06 | [0.98 - 1.14] | 28 | 1.04 | [0.97 - 1.13] | 29 |
| Oostende-city | 1.02 | [0.95 - 1.10] | 29 | 0.92 | [0.85 - 0.99] | 40 |
| Arlon-city | 1.02 | [0.88 - 1.18] | 30 | 1.13 | [0.97 - 1.31] | 25 |
| Verviers-other | 1.02 | [0.95 - 1.09] | 31 | 1.07 | [1.00 - 1.14] | 26 |
| Antwerp-city | 0.99 | [0.95 - 1.03] | 32 | 0.92 | [0.88 - 0.95] | 39 |
| Liège-other | 0.97 | [0.91 - 1.04] | 33 | 1.04 | [0.97 - 1.11] | 32 |
| Dendermonde | 0.97 | [0.92 - 1.02] | 34 | 0.96 | [0.91 - 1.01] | 36 |
| Oudenaarde | 0.95 | [0.89 - 1.02] | 35 | 0.98 | [0.92 - 1.05] | 34 |
| Aalst-other | 0.95 | [0.90 - 1.00] | 36 | 0.98 | [0.93 - 1.03] | 35 |
| Oostende-other | 0.93 | [0.85 - 1.02] | 37 | 0.90 | [0.83 - 0.99] | 41 |
| Nivelles | 0.93 | [0.89 - 0.97] | 38 | 1.05 | [1.01 - 1.10] | 28 |
| Ghent-city | 0.91 | [0.87 - 0.96] | 39 | 0.89 | [0.85 - 0.93] | 43 |
| Kortrijk-city | 0.89 | [0.83 - 0.95 ] | 40 | 0.93 | [0.86 - 0.99] | 38 |
| Halle-Vilvoorde-city | 0.88 | [0.84 - 0.92] | 41 | 0.99 | [0.95 - 1.03] | 33 |
| Diksmuide-veurne | 0.88 | [0.81 - 0.94] | 42 | 0.84 | [0.78 - 0.90] | 51 |
| Mechelen-city | 0.87 | [0.81 - 0.94] | 43 | 0.89 | [0.83 - 0.97] | 42 |
| Roeselare-city | 0.85 | [0.77 - 0.94] | 44 | 0.84 | [0.76 - 0.92] | 53 |
| Halle-Vilvoorde-other | 0.85 | [0.81 - 0.89] | 45 | 0.96 | [0.91 - 1.00] | 37 |
| Ieper | 0.84 | [0.78 - 0.91] | 46 | 0.83 | [0.77 - 0.89] | 58 |
| Eeklo | 0.84 | [0.77 - 0.91] | 47 | 0.82 | [0.75 - 0.89] | 60 |
| Bruges-city | 0.83 | [0.78 - 0.89] | 48 | 0.85 | [0.79 - 0.91] | 48 |
| Tielt | 0.83 | [0.76 - 0.90] | 49 | 0.87 | [0.80 - 0.94] | 47 |
| Kortrijk-other | 0.83 | [0.78 - 0.88] | 50 | 0.87 | [0.82 - 0.93] | 45 |
| Sint-Niklaas-city | 0.82 | [0.75 - 0.90] | 51 | 0.82 | [0.75 - 0.90] | 59 |
| Turnhout-city | 0.82 | [0.74 - 0.91] | 52 | 0.83 | [0.75 - 0.92] | 57 |
| Verviers-German speaking | 0.82 | [0.73 - 0.91] | 53 | 0.87 | [0.78 - 0.97] | 46 |
| Sint-Niklaas-other | 0.81 | [0.76 - 0.86] | 54 | 0.84 | [0.79 - 0.89] | 52 |
| Mechelen-other | 0.81 | [0.78 - 0.85] | 55 | 0.83 | [0.79 - 0.88] | 54 |
| Ghent-other | 0.81 | [0.77 - 0.85] | 56 | 0.88 | [0.84 - 0.92] | 44 |
| Roeselare-other | 0.81 | [0.74 - 0.88] | 57 | 0.83 | [0.76 - 0.90] | 56 |
| Tongeren | 0.80 | [0.75 - 0.85] | 58 | 0.83 | [0.78 - 0.88] | 55 |
| Hasselt-city | 0.79 | [0.73 - 0.87] | 59 | 0.85 | [0.78 - 0.93] | 49 |
| Bruges-other | 0.78 | [0.74 - 0.83] | 60 | 0.81 | [0.76 - 0.86] | 63 |
| Leuven-other | 0.77 | [0.74 - 0.81] | 61 | 0.84 | [0.81 - 0.88] | 50 |
| Hasselt-other | 0.77 | [0.73 - 0.81] | 62 | 0.80 | [0.76 - 0.84] | 65 |
| Leuven-city | 0.76 | [0.70 - 0.82] | 63 | 0.81 | [0.75 - 0.88] | 61 |
| Turnhout-other | 0.73 | [0.69 - 0.76] | 64 | 0.76 | [0.73 - 0.80] | 67 |
| Maaseik | 0.72 | [0.68 - 0.76] | 65 | 0.78 | [0.73 - 0.83] | 66 |
| Antwerp-urban agglomeration | 0.71 | [0.68 - 0.75] | 66 | 0.81 | [0.77 - 0.85] | 62 |
| Genk | 0.70 | [0.61 - 0.80] | 67 | 0.73 | [0.64 - 0.84] | 68 |
| Antwerp-other | 0.70 | [0.67 - 0.74] | 68 | 0.80 | [0.76 - 0.85] | 64 |
| **BIC** | 565050.47 | | | 547715.35 | | |
| **Pseudo-R2** | 0.032 | | | 0.0623 | | |
